# Phenolic compounds, flavonoids, lipids and antioxidant potential of apricot (*Prunus armeniaca* L.) pomace fermented by two filamentous fungal strains in solid state system

**DOI:** 10.1186/s13065-017-0323-z

**Published:** 2017-09-21

**Authors:** Francisc Vasile Dulf, Dan Cristian Vodnar, Eva-Henrietta Dulf, Adela Pintea

**Affiliations:** 1 Department of Environmental and Plant Protection, University of Agricultural Sciences and Veterinary Medicine Cluj-Napoca, Cluj-Napoca, Romania; 2 Department of Food Science and Technology, University of Agricultural Sciences and Veterinary Medicine Cluj-Napoca, Cluj-Napoca, Romania; 3 Faculty of Automation and Computer Science, Technical University of Cluj-Napoca, Cluj-Napoca, Romania; 4 Faculty of Veterinary Medicine, University of Agricultural Sciences and Veterinary Medicine Cluj-Napoca, Cluj-Napoca, Romania

**Keywords:** Solid-state fermentation, *Aspergillus niger*, *Rhizopus oligosporus*, Apricot pomace, Polyphenols, Antioxidant activity

## Abstract

**Background:**

The use of agricultural and food by-products is an economical solution to industrial biotechnology. The apricot press residues are abounding by-products from juice industry which can be used as substrates in solid state fermentation process (SSF), thus allowing a liberation and increase of content from various biomolecules with high added value.

**Methods:**

The evolutions of phenolic levels (by colorimetric assays and high performance liquid chromatography, HPLC–MS) and antioxidant activities (by DPPH assay) during SSF of apricot pomaces with *Aspergillus niger* and *Rhizopus oligosporus* were investigated. The changes in fatty acid compositions of oils in apricot kernels during SSFs were also analyzed by gas chromatography (GC–MS).

**Results:**

The results showed that the levels of total phenolics increased by over 70% for SSF with *R. oligosporus* and by more than 30% for SSF with *A. niger*. A similar trend was observed in the amounts of total flavonoids (increases of 38, and 12% were recorded for SSF by *R. oligosporus* and *A. niger*, respectively). Free radical scavenging capacities of methanolic extracts were also significantly enhanced. The main phenolic compounds identified through HPLC–MS in fermented apricot press residues were chlorogenic acid, neochlorogenic acid, rutin, and quercetin 3-acetyl- glucoside. This work also demonstrated that the SSF with filamentous fungal strains not only helped in higher lipid recovery from apricot kernels, but also resulted in oils with better quality attributes (high linoleic acid content).

**Conclusion:**

The utilization of apricot by-products resulting from the juice industry as waste could provide an extra income and at the same time can help in solving solid waste management problems

**Graphical abstract:**
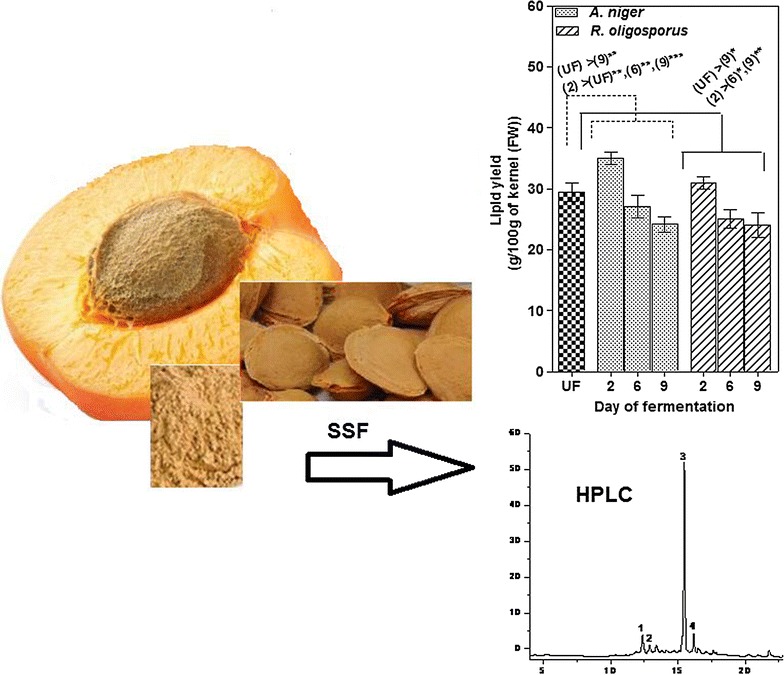
Changes in phenolic compositions, antioxidant activities and total lipid contents during solid state fermentation (SSF) of apricot pomaces from juice industry with *Aspergillus niger* and *Rhizopus oligosporus*

## Introduction

In the past few years there has been a renewed interest in re-evaluating the efficient and environmentally rational utilization or finding alternative uses for natural, renewable resources such as the agro-industrial processing lignocellulosic wastes.

Many studies have shown that important amounts of lignocellulosic biomass can potentially be converted into different high value products including bio-fuels, health promoting biomolecules, and inexpensive energy sources for microbial fermentation and enzyme production [1]. Inadequate collection and improper disposal of these agro-industrial by-products may generate significant environmental and ecological problems. Moreover, the direct disposal of these wastes into the environment, especially those originating from the fruit processing industry (from alcoholic and non-alcoholic beverages industry) leads to a significant loss of biomass which could be useful in the production of various value added metabolites [3].

The fruits of apricot (*Prunus armeniaca* L.) are characterized by high contents of nutrients and phenolic compounds such as neochlorogenic and chlorogenic acids, proanthocyanidin dimers and trimers, several quercetin and kaempferol glycosides, and cyanidin 3-glucoside as the main pigments [4]. The phytochemical composition of stone-fruits strongly depends on the cultivars and on fruit parts (skin and flesh) [5]. Many studies have demonstrated that the phenolic compounds possess a wide range of health benefits, such as free-radical scavenging property, anticancer activity, prevention of coronary heart diseases and antiviral properties [6–12].

Large amounts of fruit residues resulting from the pressing of stone fruits (such as apricots) are available in most countries of the world. These residues, called pomaces, are mostly composed of fruit skins, pulp and seeds, and are considered as waste of no value. In the available literature there are few references on polyphenol composition of apricot by-products. Although the potential of apricot as sources of different phytochemicals seems clear, there is little information available concerning the strategies for the liberation and extraction of the bioactive molecules from the vegetable matrix. The majority of the phenolics are mostly found in plants in conjugated form principally, with one or more sugar residues linked to hydroxyl groups [13]. These conjugations reduce their ability to function as good antioxidants. The enzymatic hydrolysis of conjugated polyphenols with carbohydrate degrading enzymes produced by filamentous fungal strains during the SSF can be an attractive means of increasing the amounts of free phenolics in pomaces used as substrates in the fermentation processes [14].

Solid-state fermentation is defined as a microbial culture that develops on moist substrates in the absence (or near absence) of free water [3]. The substrates must contain sufficient moisture to allow the microbial growth and metabolism. The selection of a suitable microorganism is one of the most important criteria in solid state bioprocessing. There are various factors that affect the SSF process and these vary from process to process depending upon the type of substrates and the microorganisms used, and also on the scale of the process. Filamentous fungi are the most suitable with highest adaptability for solid-state bioprocessing systems, being able to produce high quantities of enzymes with high scientific and commercial values [15].

*Aspergillus niger* and *Rhizopus oligosporus* are two filamentous fungi which have been used in many SSF studies, due to their ability to synthesize many food grade enzymes (such as cellulase, pectinase, protease, etc.) with broad substrate specificity, and low-pH and high temperature stability that have significant role in the hydrolysis of phenolic conjugates [16].

To the best of our knowledge, this is the first work that uses the apricot fruit by-products as support in SSF for the production of value-added compounds. Therefore, the aim of this study was to evaluate the changes in phenolic compositions and antioxidant activity by SSF of apricot pomaces (fruit skins, pulp) (from juice industry) with *A. niger* and *R. oligosporus.* Moreover, the effect of fermentation time on the total lipid content in solid state fermented apricot kernels was also studied.

## Materials and methods

### Raw material and chemicals

The stones from fully ripened apricot (*Prunus armeniaca* L.) fruits were removed and individually broken to obtain the intact kernels. The press cake residues (pomaces—composed of fruit skins and pulp) were obtained in our laboratory from de-stoned of yellow apricot fruits collected in July 2016. The pomace and kernels were dried in oven (37 °C) until complete drying, ground and stored in refrigerator before use.

Folin-Ciocalteu’s phenol reagent, sodium carbonate (Na_2_CO_3_), sodium nitrite (NaNO_2_), ammonium nitrate (NH_4_NO_3_), hydrochloric acid (HCl), aluminum chloride (AlCl_3_), sodium hydroxide (NaOH), salts for nutrient solution, glucose, acetic acid, acetonitrile, methanol, phenolic standards, DPPH (1,1-diphenyl-2 picrylhydrazyl) were purchased from Sigma-Aldrich (Steinheim, Germany). The FAMEs (fatty acid methyl esters) standard (37 component FAME Mix, SUPELCO) was purchased from Supelco (Bellefonte, PA, USA). All chemicals and reagents used in this study were of analytical grade.

### Culture medium and fermentation conditions

#### Culture medium

*Aspergillus niger* (ATCC-6275) and *Rhizopus oligosporus* (ATCC-22959) (LGC Standards GmbH, Wesel Germany) were selected as suitable fungi for SSF and were maintained on potato dextrose agar (PDA) slants and Petri plates at 4 °C [17]. The fungal spores were collected from the sporulation medium plates, inoculated into sterile distilled water, and stored in the freezer.

#### Solid-state fermentation

500 mL Erlenmeyer flasks containing 15 g solid substrates, 30 mL of a nutrient solution NaNO_3_ (4 g/L), K_2_HPO_4_ (2 g/L), MgSO_4_ (0.25 g/L), glucose (10 g/L) and NH_4_NO_3_ (1 g/L), were used for SSF. The fermentation mediums were autoclaved at 121 °C for 30 min and inoculated with spore suspension (2 × 10^7^ spores/g of solid). After being thoroughly mixed, the fermentations were conducted for 14 days at 30 °C. The experiments were performed in triplicate. During SSF, 1 g of samples of the media were taken at different time points for analysis [16, 17].

#### Extraction and analysis of phenolic compounds

The apricot pomace samples (2 g) were individually extracted three times with 20 mL of extraction mixture (hydrochloric acid/methanol/water in the ratio of 1:80:19) at 40 °C for 30 min in an ultrasonic bath [16]. The resulting dried extracts were dissolved in methanol and stored (4 °C) until analysis (total and individual phenolics, total flavonoids and antioxidant activities).

#### Total phenolics

The total phenolic amounts were determined by the Folin–Ciocalteu method [26], using a Synergy HT Multi-Detection Microplate Reader with 96-well plates (BioTek Instruments, Inc., Winooski, VT, USA). An aliquot (25 μL) of each extract was mixed with 125 μL of Folin–Ciocalteu reagent (0.2 N) and 100 μL of 7.5% (w/v) Na_2_CO_3_ solution [16]. The absorbance against a methanol blank was recorded at 760 nm. A standard curve was prepared using gallic acid and the TP content in the extract was expressed as gallic acid equivalents (GAE) in mg/100 g fresh weight (FW) of waste.

#### Total flavonoids

The total flavonoid amounts were measured according to the aluminium chloride colorimetric method developed by Zhishen et al. [26] using quercetin as reference standard, as described by Dulf et al. [17]. The absorbance was measured at 510 nm. Total flavonoid content was expressed as mg quercetin equivalent (mg QE/100 g FW).

### Analysis of individual phenolic compounds by HPLC–DAD-ESIMS (high-performance liquid chromatography-diode array detection-electro-spray ionization mass spectrometry)

The phenolic extracts were analyzed using an Agilent 1200 HPLC with DAD detector, coupled with MS detector single quadrupole Agilent 6110. The separations of phenolic compounds were performed at 25 °C on an Eclipse column, XDB C18 (4.6 × 150 mm, 5 μm) (Agilent Technologies, USA). The binary gradient consisted of 0.1% acetic acid/acetonitrile (99:1) in distilled water (v/v) (solvent A) and 0.1% acetic acid in acetonitrile (v/v) (solvent B) at a flow rate of 0.5 mL/min, following the elution program used by Dulf et al. [16]: 0–2 min (5% B), 2–18 min (5–40% B), 18–20 min (40–90% B), 20–24 min (90% B), 24–25 min (90–5% B), 25–30 min (5% B).

The phenolics were identified by comparing the retention times, UV- visible and mass spectra of unknown peaks with the reference standards. For MS fragmentation, the ESI(+) module was applied, with scanning range between 100 and 1000 *m/z*, capillary voltage 3000 V, at 350 °C and nitrogen flow of 8 L/min. The eluent was monitored by DAD, and the absorbance spectra (200–600 nm) were collected continuously in the course of each run. The flavonols were detected at 340 nm [17]. Data analysis was performed using Agilent ChemStation Software (Rev B.04.02 SP1, Palo Alto, California, USA). The chlorogenic and neochlorogenic acids were expressed in mg chlorogenic acid/100 g FW of substrate and flavonol glycosides were calculated as equivalents of rutin (mg rutin/100 g FW of substrate).

### DPPH free radical scavenging assay

The antioxidant activity of the obtained phenolic extracts were determined by DPPH radical scavenging assay, using the method described by Dulf et al. [17]. The percentage inhibition (I%) was calculated as [1 − (test sample absorbance/blank sample absorbance)] × 100.

### Oil extraction and fatty acid analysis

The non- and fermented (after 2, 6 and 9 days of SSF) apricot kernels (5 g) were extracted with 60 mL solution of chloroform: methanol (2:1, v/v) [17]. The oil contents were determined gravimetrically. An aliquot (10–15 mg) of each lipid extract was transesterified into FAMEs using the acid-catalyzed method [9] and analyzed by gas chromatography–mass spectrometry (GC–MS) using a previously described protocol [17]. A GC–MS (PerkinElmer Clarus 600 T GC–MS (PerkinElmer, Inc., Shelton, CT, USA)) equipped with a Supelcowax 10 capillary column was used (60 m × 0.25 mm i.d., 0.25 μm film thickness; Supelco Inc., Bellefonte, PA, USA). The column temperature was programmed from 140 to 220 °C at a rate of 7 °C/min and held for 23 min. Helium was used as carrier gas at a constant flow rate of 0.8 mL/min. The mass spectra were recorded in EI (positive ion electron impact) mode. The mass scans were performed from *m/z* 22 to 395. Identification of fatty acids was carried out by comparing their retention times with those of known standards and the generated mass spectral data with those of the NIST library (NIST MS Search 2.0).

Quantification of the fatty acids was achieved by the comparison of peak areas with internal standard (nonadecanoic acid, Sigma, Steinheim, Germany) which was added to the samples (200 μg) prior to methylation, without application of any correction factor. Fatty acid compositions of oils in apricot kernels were expressed as weight percentages of the total fatty acids.

### Statistical analysis

All tests were conducted in triplicate and the results were presented as mean ± standard deviation (SD). Correlations among the antioxidant activity and phenolics were calculated using Pearson’s correlation. Statistical analyses were performed by Student’s t-test and ANOVA (repeated measures ANOVA; Tukey’s Multiple Comparison Test; GraphPad Prism Version 5.0, Graph Pad Software Inc., San Diego, CA). Differences between means at the 5% level were considered statistically significant.

## Results and discussion

### Total phenolic and flavonoid contents. HPLC–MS analysis of individual phenolic compounds

**Fig. 1:**
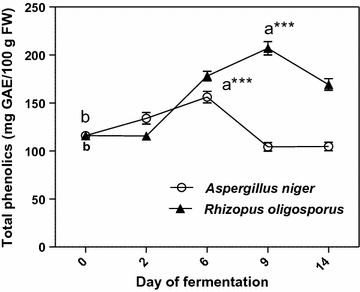
Total phenolic content of extracts from solid state fermented apricot pomaces. Values are mean ± SD of triplicate determinations and different letters (a, b) indicate significant differences (p < 0.05) (paired t-test)

The total phenolic amounts determined by Folin-Ciocalteu procedure showed a similar increasing trend over the first 6 days of solid-state fermentation for both filamentous fungal strains. This trend has continued only for fermentation with *R. oligosporus* until day 9, after that the total soluble phenolics sharply decreased for the remaining days of SSF (Fig. 1).

The increase in total phenolic content was higher when *R. oligosporus* was used for fermentation (78%-day 9), compared to *A. niger* (34%-day 6). These increases of measurable free phenolics contents could be attributed to the fungal-derived β-glucosidases which can hydrolyze β-glucosidic bonds, mobilizing the free phenolic compounds to react with the Folin–Ciocalteau reagent [14]. Similar tendencies in phenolic contents were also observed in our previous studies [16, 17]. The free phenolics amounts showed significant decrease in the second part of fermentations (Fig. 1) which could be due to the polymerization and lignification of the released free phenolics by lignifying and tannin forming peroxidases, activated in response to the stress induced on the microorganism due to the nutrient deficiencies [18].

**Fig. 2:**
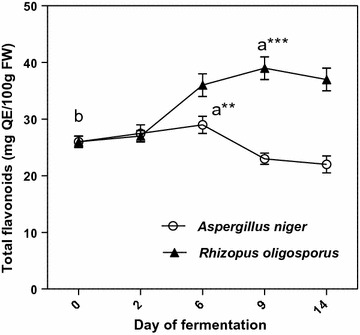
Total flavonoids in extracts from solid state fermented apricot pomaces. Values are mean ± SD of triplicate determinations and different letters (a, b) indicate significant differences (p < 0.05) (paired t-test)

The total flavonoid contents of solid-state processed apricot by-products showed similar trends as total phenolic amounts (Fig. 2).

In the first 6 days of fermentation with *A. niger*, and after 9 days of SSF by *R. oligosporus*, significant increases were observed in flavonoid contents until the maximum yields of 29 mg QE/100 g pomace, FW-by *A. niger* and 36 mg QE/100 g pomace, FW-by *R. oligosporus*, respectively (from the initial value of 26 mg QE/100 g FW). An important increase in the levels of total flavonoids was also observed by Lin et al. [19] in *Aspergillus*-fermented litchi pericarp. According to Ruiz et al. [4], the total polyphenol and flavonoid contents in apricot strongly depend on varieties.

**Fig. 3:**
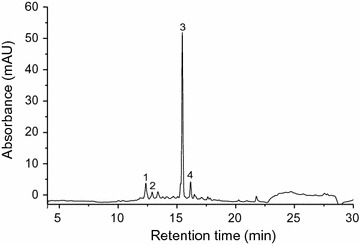
HPLC chromatogram (detected wavelength at 340 nm) of *R. oligosporus* fermented apricot pomace (9th day of SSF): (1) 3-caffeoylquinic acid; (2) 5-caffeoylquinic acid; (3) quercetin-3-rutinoside; (4) Quercetin-3(6″acetyl-glucoside)

The quantities of the main phenolics in the extracts of apricot pomaces were determined during solid-state fermentations (Table 1), using HPLC–DAD-MS. All samples contained four dominant phenolics: two cinnamic acids (3-caffeoylquinic and 5-caffeoylquinic acids) and two flavonols (quercetin-3-rutinoside and quercetin-3(6″acetyl-glucoside)) (Fig. 3).

**Table 1:** Mean phenolic contents (mg/100 g FW) of apricot pomaces during solid-state fermentation

Phenolics ([M + H] + ion, fragments	Cinnamic acids		Flavonols	
	3-CQA (355, 181)	5-CQA (355, 181)	Q-3-rut (611, 303)	Q-3-ac-gluc* (517,303)
*Aspergillus niger*				
FD				
0	7.81 ± 0.31^a^	15.14 ± 0.61^a^	16.50 ± 0.66^a^	5.71 ± 0.23^a^
2	5.23 ± 0.21^b^	12.17 ± 0.49^c^	15.65 ± 0.63^b^	4.81 ± 0.19^b^
6	4.93 ± 0.20^c^	12.65 ± 0.51^b^	15.61 ± 0.62^b^	4.26 ± 0.17^c^
9	4.58 ± 0.18^d^	11.78 ± 0.47^d^	14.50 ± 0.58^c^	4.15 ± 0.17^d^
14	4.23 ± 0.17^e^	11.26 ± 0.45^e^	14.13 ± 0.57^d^	4.07 ± 0.16^e^
*Rhizopus oligosporus*				
0	7.81 ± 0.31^a^	15.14 ± 0.61^a^	16.50 ± 0.66^b^	5.71 ± 0.23^a^
2	5.06 ± 0.20^e^	10.21 ± 0.41^e^	15.69 ± 0.63^d^	4.70 ± 0.19^d^
6	5.76 ± 0.23^d^	11.74 ± 0.47^d^	15.98 ± 0.64^c^	4.89 ± 0.20^c^
9	6.59 ± 0.26^b^	13.48 ± 0.54^b^	18.17 ± 0.73^a^	5.15 ± 0.21^b^
14	5.98 ± 0.24^c^	12.39 ± 0.50^c^	16.58 ± 0.66^b^	4.59 ± 0.18^e^

Values (mean ± SD, n = 3) in the same column with different letters (a–e) significantly differ (*p* < 0.05) (ANOVA “Tukey’s Multiple Comparison Test”). *FD* fermentation day, *3-CQA* 3-caffeoylquinic acid (neochlorogenic acid), *5-CQA* 5-caffeoylquinic acid (chlorogenic acid), *Q-3-rut* quercetin-3-rutinoside (rutin), *Q-3-ac-gluc* Quercetin-3(6″acetyl-glucoside) * Tentative identification

These phenolic profiles were in general agreement with the study of Ruiz et al. [4] on phenolic composition in peels of different apricot varieties. Chlorogenic acid and rutin were the major phenolics in both processed pomaces (Table 1). Overall, the SSF with both fungal strains had significant effect (p < 0.05) on evolution of phenolic amounts. In general, a decrease of the individual phenolic concentrations in all samples (excepting quercetin-3-rutinoside, as main flavonol in fermented samples with *R. oligosporus*) was observed (Table 1).

The usefulness of by-products from the beverage industry is still underestimated. To our knowledge this is the first study investigating the variation of the amounts of phenolic compounds in apricot pomaces from the beverage industry in correlation to fermentation days in solid-state system with these two filamentous fungi.

### Antioxidant activity

**Fig. 4:**
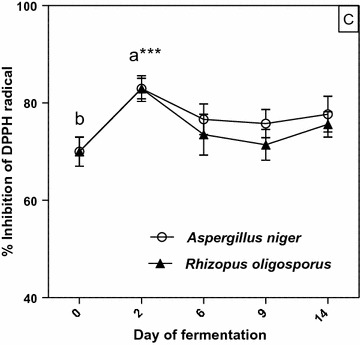
Free radical scavenging activity (DPPH assay) of phenolics in extracts from solid state fermented apricot pomaces. Values are mean ± SD of triplicate determinations and different letters (a, b) indicate significant differences (p < 0.05) (paired t-test)

The evolution of the antioxidant potential of methanol extracts from solid state fermented apricot by-products were measured using the DPPH radical scavenging assay and the results are presented in Fig. 4. The DPPH assay is widely used to determine antioxidant activity of phenolic compounds in natural plant extracts. This assay is based on the capacity of stable free radicals of DPPH to react with hydrogen donors.

After each SSF process, statistically significant increases in antioxidant activity levels (*p* < 0.05) of the analyzed extracts were registered. The antioxidant capacity increased by over 18% for both fungal fermentations by day 2 compared with the initial value (%I = 70) before gradually decreasing for the remaining period of growth.

**Fig. 5:**
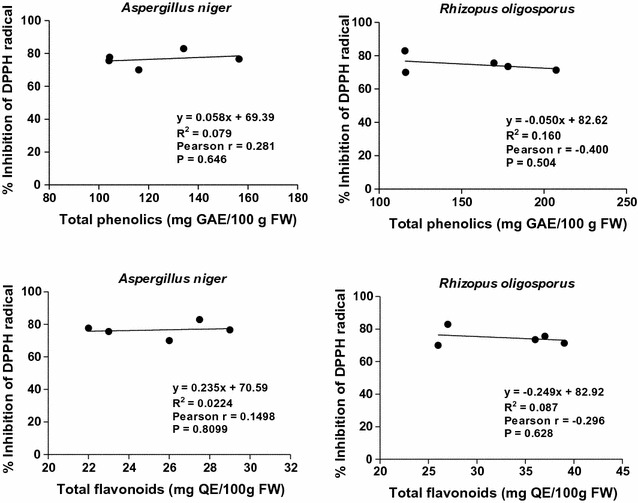
Correlation coefficients between antioxidant activity (DPPH) and total phenolics and total flavonoids in the extracts of solid-state fermented apricot press residues

**Fig. 6:**
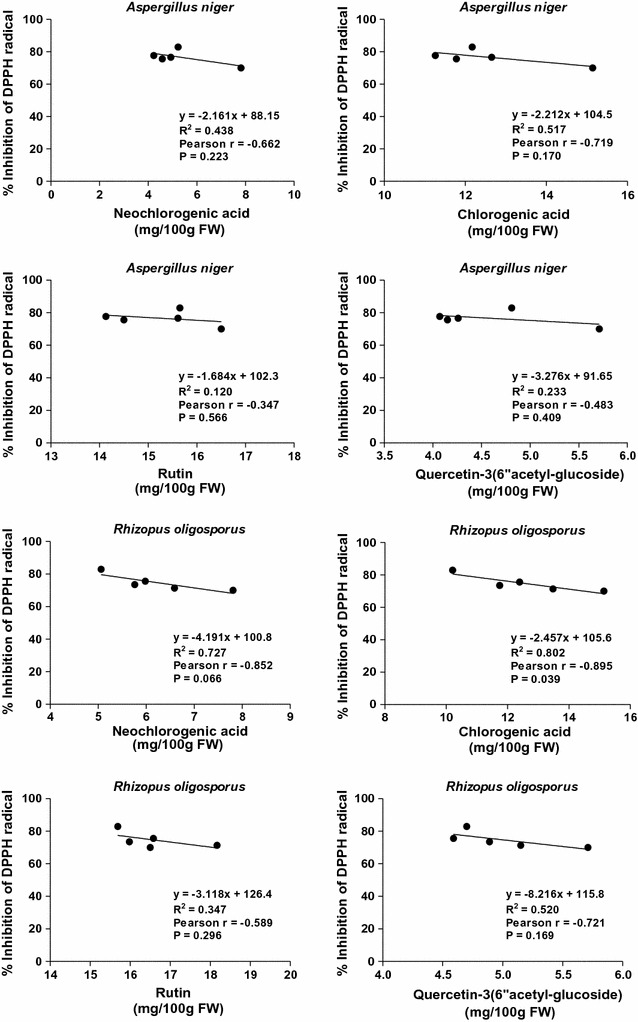
Correlation coefficients between antioxidant activity (DPPH) and the main individual phenolics in the extracts of solid-state fermented apricot press residues

In the case of SSF with *A. niger*, weak positive (0.1 < *r* < 0.3), but statistically not significant (*p* > 0.05) correlations were found between antioxidant capacity (determined by DPPH assay) and total phenolic and flavonoid contents (Fig. 5). It is also worth to mention that the values obtained for SSF with *R. oligosporus* correlated negatively (*r* < 0, *p* > 0.05) (Fig. 5). Moreover, the results presented in Fig. 6 also revealed a negative relationship between the concentrations of individual phenolic compounds and antioxidant activity of the methanolic extracts.

These correlation analyses suggested that the individual phenolic compounds could not be the key constituents responsible for the free radical scavenging activity of studied fermented samples.

These findings are mainly in agreement with our previous observations [16, 17] and with reported data from other authors [3, 18]. In all these reports (on different bio-processed agro-food wastes and cereals), weak correlations between polyphenolic contents and antioxidant capacities were found. This may be caused by the polymerization of phenolic monomers due to the stress induced on the fungus in certain phases of its growth. Many studies have shown that increasing degree of polymerization enhances the effectiveness of phenolics against a variety of free radical species due to the increment of the hydroxyl groups in addition to the extensive conjugations between double bonds and carbonyl groups from their structures [20, 21].

### Changes in lipid and fatty acid compositions during SSF of the apricot kernels by *A. niger* and *R. oligosporus*

**Fig. 7:**
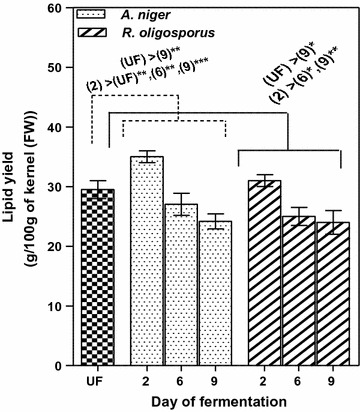
The time course of oil production by *Aspergillus niger* and *Rhizopus oligosporus* strains in apricot kernels during SSF. Results are given as mean ± SD (n = 3); *p < 0.05, **p < 0.01, ***p < 0.001 (repeated measures ANOVA “Tukey’s Multiple Comparison Test”). *UF* unfermented

The data on oil content of apricot kernels processed with *A. niger* and *R. oligosporus* are shown in Fig. 7. Both fungal strains increased the total lipid content until the second day of fermentation.

The unfermented apricot kernels showed a fat content of 29.50 g/100 g of kernel (FW), which is close to the values already reported in the literature [22]. The extracted lipids increased significantly (p < 0.05) by 18.64% from the initial value for the solid state fermented kernels with *A. niger* whereas for *R. oligosporus* the evolution of these biomolecules was statistically insignificant (p > 0.05) (1.50%). It can be concluded that *A. niger* has a better lipogenic effect than *R. oligosporus* when grown on apricot kernels.

Recent studies have shown that the enzymes (cellulase, pectinase, protease, etc.) produced by the filamentous fungi in solid state system have a determining role in degradation of oil seeds cell wall, leading to the release of most of the lipids (generally bound to proteins or to the polyglucides) enmeshed in cellular structures [23]. The researchers have reported maximum enzyme activity until 48–72 h of SSF, depending on culture conditions (available fermentable sugars (carbon source), carbon to nitrogen (C:N) ratio of the fermentation medium, temperature, pH, etc.) after which the enzyme production had stabilized or decreased. These observations are in agreement with our findings regarding the dynamics of the lipid yields presented in Fig. 7, with the maximum oil amounts in the 2nd day of SSF.

The changes in fatty acid compositions of oils in apricot kernels during SSFs with *A. niger* and *R.oligosporus* are shown in Table 2. The predominant fatty acids in all processed samples were oleic acid (C18:1n − 9), linoleic acid (C18:2n − 6), and palmitic acid (C16:0). The SSF processes have caused statistically significant (p < 0.05) decreases of the palmitic (C16:0) and stearic (C18:0) acids, and a substantial increase (p < 0.05) in the content of linoleic acid (C18:2(n − 6)) and oleic acid (C18:1(n − 9)), respectively (Table 2). Moreover, the studied oils are characterized by high levels of unsaturated fatty acids (mono-(MUFAs) and polyunsaturated fatty acids (PUFAs)). The elevated levels of MUFAs from the analyzed apricot kernel oils are comparable to those of MUFA-rich vegetable oils, such as rapeseed, avocado, olive etc. [24].

**Table 2:** Fatty acid compositions (%) (determined by GC–MS) of total lipids produced by *Aspergillus niger* and *Rhizopus oligosporus* in solid-state fermentation (day 2) of apricot kernels

Fatty acids (%)	Samples					
	Unfermented		SSF with *Aspergillus niger*		SSF with *Rhizopus oligosporus*	
	Mean	SD	Mean	SD	Mean	SD
16:0	7.31^a^	0.29	3.80^c^	0.15	4.80^b^	0.19
16:1 (n − 9)	0.05	0.01	0.04	0.01	0.04	0.01
16:1 (n − 7)	1.95	0.08	0.83	0.03	0.83	0.03
17:0	0.04	0.01	0.06	0.01	0.06	0.01
17:1 (n − 9)	0.11	0.02	0.11	0.02	0.11	0.02
18:0	2.81^a^	0.11	1.00^c^	0.04	1.15^b^	0.05
18:1 (n − 9)	51.46^c^	2.06	54.10^a^	2.16	53.90^b^	2.16
18:1 (n − 7)	3.58	0.14	3.65	0.15	3.70	0.15
18:2 (n − 6)	32.30^c^	1.29	36.20^a^	1.45	35.21^b^	1.41
18:3 (n − 3)	0.14	0.02	0.07	0.01	0.09	0.01
20:0	0.16	0.02	0.06	0.01	0.04	0.01
20:1 (n − 9)	0.10	0.02	0.08	0.01	0.07	0.01
SFAs	10.32^a^	0.41	4.92^c^	0.20	6.05^b^	0.24
MUFAs	57.25^c^	2.29	58.81^a^	2.35	58.65^b^	2.34
PUFAs	32.44^c^	1.30	36.27^a^	1.45	35.30^b^	1.41
n − 3 PUFAs	0.14^a^	0.02	0.07^c^	0.01	0.09^b^	0.01
n − 6 PUFAs	32.30^c^	1.29	36.20^a^	1.45	35.21^b^	1.40

Values are the means of three measurements (n = 3). Different superscript letters (a–c) in the same row indicate significant differences (p < 0.05) among fatty acids of the unfermented and fermented substrates (ANOVA “Tukey’s Multiple Comparison Test”). C16:0 palmitic; C16:1(n − 9) cis-7 hexadecenoic; C16:1(n − 7) palmitoleic; C17:0 margaric; C17:1(n − 9) heptadecenoic; C18:0, stearic; C18:1(n − 9), oleic; C18:1(n − 7), vaccenic; C18:2(n − 6), linoleic; C18:3(n − 3), α-linolenic; C20:0, arachidic; C20:1(n − 9), 11-eicosenoic; *SFAs* saturated fatty acids; *MUFAs* monounsaturated fatty acids; *PUFAs* polyunsaturated fatty acids

The evolutions of the major fatty acids during the SSF are in agreement with the previously reported data, which demonstrated that the filamentous fungi are able to produce lipids with considerable proportions of unsaturated fatty acids [25].

## Conclusions

The present work showed that the enrichment of apricot pomaces with phenolic compounds can be achieved by solid-state bioprocessing using food grade fungi. Total phenolic contents increased by over 78% for SSF with *R. oligosporus* and by more than 30% for SSF with *A. niger*. The total flavonoid levels showed similar tendencies with the total phenolics. HPLC analysis showed a relative decrease in the amounts of each phenolic compound during the SSF processes. The antioxidant potential determined by DPPH radical scavenging assay increased significantly (> 18%) over the course of growth.

This work also demonstrated that the solid-state fermentation with filamentous fungal strains not only helped in higher lipid recovery from apricot kernels, but also resulted in oils with better quality attributes (high linoleic acid content). The high lipid content of apricot kernels, comparable to oleaginous seeds, such as rapeseed or sunflower, makes them suitable for commercial oil production.

This research may potentially provide the basis for a sustainable process of integrated exploitation of apricot by-products as potential, cheap, and easily available sources of high value phytochemicals for the pharmaceutical and food industries.
